# Quality of Life and Its Predictive Factors Among Healthcare Workers After the End of a Movement Lockdown: The Salient Roles of COVID-19 Stressors, Psychological Experience, and Social Support

**DOI:** 10.3389/fpsyg.2021.652326

**Published:** 2021-04-09

**Authors:** Luke Sy-Cherng Woon, Nor Shuhada Mansor, Mohd Afifuddin Mohamad, Soon Huat Teoh, Mohammad Farris Iman Leong Bin Abdullah

**Affiliations:** ^1^Department of Psychiatry, Universiti Kebangsaan Malaysia Medical Centre, Kuala Lumpur, Malaysia; ^2^Lifestyle Science Cluster, Advance Medical and Dental Institute, Universiti Sains Malaysia, Pulau Pinang, Malaysia

**Keywords:** quality of life, healthcare workers, COVID-19-related stressors, psychological sequelae, perceived social support, Malaysia

## Abstract

Although healthcare workers play a crucial role in helping curb the hazardous health impact of coronavirus disease 2019 (COVID-19), their lives and major functioning have been greatly affected by the pandemic. This study examined the effects of the COVID-19 pandemic on the quality of life (QoL) of Malaysian healthcare workers and its predictive factors. An online sample of 389 university-based healthcare workers completed questionnaires on demographics, clinical features, COVID-19-related stressors, psychological experiences, and perceived social support after the movement lockdown was lifted. All domains of QoL were within the norms of the general population except for social relationship QoL, which was lower than the norm. Multiple linear regression analysis indicated that COVID-19-related stressors (e.g., stress due to annual leave being frozen, loss of daily routine, and frequent exposure to COVID-19 patients) and psychological sequelae (e.g., greater severity of depression, anxiety, and stress) predicted lower QoL. Conversely, greater perceived social support from friends and significant others predicted higher QoL. Clinical and demographic characteristics predicted QoL to a lesser extent: A history of pre-existing medical illness was associated only with lower physical health QoL, whereas older age and being single, divorced, or widowed were only predictive of higher environmental QoL. Efforts to enhance QoL among healthcare workers in response to the pandemic should focus on mitigating COVID-19-related stressors and psychological sequelae and facilitating social support.

## Introduction

Coronavirus disease 2019 (COVID-19), caused by severe acute respiratory syndrome coronavirus 2 (SARS-CoV-2), was first detected in Wuhan, China, in late 2019, and it has since spread globally. The rapid spread of the virus, transmitted primarily by human-to-human contact, drove the World Health Organization to classify it as a pandemic in March 2020. Given the virus's mode of transmission, countermeasures have been imposed to break the chain of infection, including social distancing to minimize the spread from unknown sources, quarantine to safeguard against possible infection, and isolation to limit the spread from known sources (Saltzman et al., 2020). Notably, the effect of this pandemic is not limited to physical health but also relates to psychological and social functioning, as well as the safety of the surrounding environment. For instance, mounting evidence suggests a high prevalence of depression, anxiety, stress, and trauma affecting people placed under strict measures to contain the disease (Gao et al., 2020; Huang and Zhao, 2020; Lai et al., 2020; Mazza et al., 2020; Odriozola-González et al., 2020; Özdin and Özdin, 2020). Social functioning is also severely affected by the pandemic because of social distancing; in this sense, reduction in social capital (El-Zoghby et al., 2020; Xiao et al., 2020a,b), loss of social routine, loneliness due to isolation (Gonçalves et al., 2020), and social boycott due to the stigma of infection (Mamun and Griffiths, 2020) are some examples of impairment in social relationships during this unprecedented time.

In observing the safety measure to contain the virus, the response to this new norm may be unique to healthcare workers. When compared with the public, healthcare workers are highly susceptible to negative psychological responses (Li et al., 2020a) exacerbated by the risk of contact with infected patients (Lai et al., 2020; Lu et al., 2020; Zhang et al., 2020a). With the lack of evidence-based practice related to COVID-19 patient management, the infection has an unusual tendency to arouse fear and subsequent ineffective psychological and social response adaptation, threatening optimal quality of life (QoL). A study on the QoL of healthcare workers during the Ebola outbreak in Africa reported a significant decrease in physical health and psychological QoL (Jones et al., 2020), indicating the need to evaluate the QoL of healthcare workers during the COVID-19 pandemic. In fact, a small-scale online survey on the QoL of healthcare workers in the Netherlands (*n* = 52) indicated that the self-reported QoL of healthcare workers was significantly lower during the peak of COVID-19 when compared with their QoL prior to the onset of the COVID-19 pandemic (Jeyaratnam et al., 2020).

Previous research has pointed out the lack of studies on how the COVID-19 pandemic affects people's professional and personal lives, and including the QoL of healthcare workers (Testoni et al., 2021). Melo-Oliveira et al. (2020) conducted a systematic review examining the effects of COVID-19 and presented evidence of impaired QoL among participants across multiple nations (Italy, China, Saudi Arabia, and Vietnam). However, it is important to note that the review, which includes publications up to May 2020, detected only a single study investigating healthcare workers' QoL (Amerio et al., 2020). Perceived QoL concerns not only the absence of physical disease but also the subjective evaluation of an individual's psychological, social, and environmental condition (World Health Organization, 1996). Although there have been more studies on QoL of healthcare workers published after Melo-Oliveira et al. (2020) systematic review, and these studies have reported a significant proportion of healthcare workers with low health-related QoL affected by depression, anxiety, stress, poor self-perceived mental health status, insomnia, and working in COVID-19-designated hospitals during the COVID-19 pandemic (Amerio et al., 2020; An et al., 2020; Çelmeçe and Menekay, 2020; Manh Than et al., 2020; Stojanov et al., 2020; Suryavanshi et al., 2020), little is known about how the COVID-19 pandemic influences the different domains of QoL (physical health, psychological, social relationship, and environmental QoL). Furthermore, even less is understood about the associated factors that worsen or improve QoL, especially among those working in high-risk clinical settings during this pandemic. Moreover, data on how different sources of social support and COVID-19-related stressors affect the QoL of healthcare workers is lacking and not investigated in previous studies.

In research conducted in Malaysia, perceived inadequacy of social support received at work, suffering from some medical illnesses, long working hours without leave (more than 60 h per week), irregular spirituality routines, and direct involvement with COVID-19 patients leading to frequent exposure significantly predicted higher odds of burnout among healthcare workers in this country during the COVID-19 pandemic (Roslan et al., 2021). Higher levels of burnout are associated with lower general QoL and psychological QoL among healthcare workers (Asante et al., 2019). In addition, having a history of mental illness contributes to poor QoL among healthcare workers (Lua et al., 2018). However, the association of these factors with QoL of healthcare workers has not been investigated during the COVID-19 pandemic.

Based on the literature, it is hypothesized that the QoL among healthcare workers during this pandemic is much worse than that of the general population in the non-pandemic state (Hawthorne et al., 2006). To address this gap in our present understanding, the present work aimed to assess the QoL of healthcare workers in response to the COVID-19 pandemic. In addition, this work explored the extent to which various factors, such as demographics, clinical aspects, COVID-19-related stressors, psychological experiences, and perceived social support, were linked to QoL.

## Materials and Methods

### Study Design and Respondents

This cross-sectional online survey was conducted from July 1–21, 2020, ~3 weeks after a nationwide movement control order (MCO) was lifted. The estimation of sample size required for the study was based on the formula for calculation of sample size needed for estimating a single mean: *N* = [(Z1-α/2 x σ)/Δ]^2^ (where *N* was the total estimated sample size; Z1-α/2 was the value represented by the desired confidence interval of 95% [95% CI], which is equal to a critical value of 1.96; σ was the standard deviation (SD), calculated as 18.1 based on the norm of the QoL in the general population, Hawthorne et al., 2006; and Δ was precision, with a value of 2.5). Hence, the estimated sample size required was 241 respondents (with an addition of 20% of sample loss). The data analyzed were partly based on the data from a cross-sectional survey of the prevalence of depression, anxiety, stress, and their associated factors among university healthcare workers in Malaysia during the COVID-19 pandemic (Woon et al., 2020). The source population was healthcare workers who worked in university hospitals and healthcare facility settings (university health clinics), which included medical staff (e.g., medical lecturers, physicians, medical officers), and allied healthcare staff (e.g., nurses, medical assistants, physiotherapists, nutritionists, radiographers, pharmacists, and assistant pharmacists). Subjects were recruited via snowball sampling; initially, medical lecturers who worked in university hospitals located in the northern states of Penang and Kelantan, as well as Klang Valley in the central part of Peninsular Malaysia, were invited to participate in the study by email. They were also asked to disseminate the invitation emails to other medical staff and allied healthcare staff in university hospitals and healthcare facility settings throughout Penang, Kelantan, and Klang Valley in a chain of referrals. Penang, Kelantan, and Klang Valley were selected as targeted sources of subject recruitment because these states are located in northern and central Peninsular Malaysia and have a high density of university healthcare workers. Moreover, Klang Valley is an urban conglomeration with a highly dense population of 8 million people, and it is made up of two regions (Selangor and Kuala Lumpur) that had the highest numbers of COVID-19 positive cases in the country during the time of data collection (World Health Organization, 2020).

University healthcare workers were selected as a subgroup of the population engaged in a multitasking job scope, where they are required to provide healthcare service to the general population and manage academic activities in their universities. Hence, it was expected to be interesting to investigate how the COVID-19 pandemic affected their QoL. Those who fulfilled all the following eligibility criteria for the study were invited to participate: (1) medical staff and allied healthcare staff who worked in university hospitals and healthcare facilities in the states of Penang and Kelantan and the Klang Valley in Peninsular Malaysia; (2) age 18 years and above; (3) literacy in Bahasa Melayu (the official language of Malaysia); and (4) no history of pre-existing psychotic disorders, bipolar mood disorder, or illicit drug use (as these may result in impaired mental capacity to answer questionnaires, e.g., psychotic features and/or cognitive impairment). Detailed information regarding the study purposes, procedures, benefits, and risks of participating in the study were provided in the invitation email.

To reduce social desirability bias, the respondents were assured anonymity and confidentiality of the information they disclosed as they did not have to disclose their name, identity card number, or passport number, nor were they asked to provide any personal identification information in the online survey. They were also told that the information they disclosed would be safely stored and that only the research team would have access to the information. The information they disclosed would be assessed as grouped data rather than individually. Informed consent to participate in the study was considered approved by the potential subjects when they completed and submitted responses to the online survey. The questionnaires were administered to the subjects via a survey administration software called Google Forms. Initially, 450 respondents answered the questionnaires online. All the respondents completed all the questionnaires because the questionnaires could not be submitted unless all items were complete. Double responses from each respondent were prevented by switching on the “limiting responses to once per person” function in Google Forms. To minimize response bias, we excluded 61 respondents from the study because they took <60% of the median time for answering questionnaires (The median time all respondents took to answer all questionnaires was 15 min). Hence, the final sample size of the study was 389 respondents. This study received approval from the Human Research Ethics Committee of Universiti Sains Malaysia (USM/JEPeM/COVID19-21) and the Medical Research Committee of the Faculty of Medicine, Universiti Kabangsaan Malaysia (UKMPPI/111/8/JEP-2020-370).

### Measures

The dependent variable in this study comprised the levels of the QoL domains of the respondents. The independent variables assessed included the demographic and clinical characteristics of respondents; severity of depression, anxiety, and stress symptoms; COVID-19-related factors; and level of social support.

#### Demographic Characteristics

Data collected included age, gender, education level, marital status, religion, and living situation. Responses for age were reported as a continuous variable. Responses for gender were grouped into male or female; marital status responses were grouped into married or single/divorced/widowed; education level was categorized as up to secondary education or up to tertiary education; religion was categorized as non-Muslim or Muslim; and living situation was grouped into living alone, living with colleagues/friends, or living with family.

#### Clinical Characteristics

Among the clinical characteristics assessed, any history of pre-existing medical illness was elicited from respondents with the question, “Have you been diagnosed with any medical illness by a doctor?” Any history of pre-existing psychiatric illness was elicited with the question, “Have you been diagnosed with any psychiatric illness by a psychiatrist?” The responses to both items were grouped into “no” vs. “yes.”

#### COVID-19-Related Factors

The COVID-19-related factors assessed in this study were based on factors associated with psychological sequelae of previous infection epidemics, such as SARS and Middle East respiratory syndrome (MERS; Hawryluck et al., 2004; Marjanovic et al., 2007; Reynolds et al., 2008; Braunack-Mayer et al., 2013; Jeong et al., 2016; Desclaux et al., 2017; Wilken et al., 2017). Worry about family members' health during the COVID-19 pandemic was assessed via the question, “Did you worry about the health of your family members during the COVID-19 pandemic?” Stress because of loss of daily routine during the COVID-19 pandemic was evaluated with the question, “Did you feel stressed because of loss of your normal daily routine during the COVID-19 pandemic?” Those who responded “yes” were further questions on disruption of which normal daily routine led them to feel stressful. Stress because of annual leave being frozen during the COVID-19 pandemic was assessed via the question, “Did you feel stressed because your annual leave was frozen during the COVID-19 pandemic?” Those who responded “yes” would have to answer further questions on what caused them to feel stress when their annual leave was frozen. Stress because of frequent exposure to COVID-19 patients was evaluated with the question, “Did you feel stressed because you were frequently exposed to COVID-19 patients as a result of your work commitment during the COVID-19 pandemic?” Those who responded “yes” were asked further questions on what caused them to feel stress when they were frequently exposed to COVID-19 patients because of their work commitment during the COVID-19 pandemic. Fear because of having physical symptoms resembling symptoms of COVID-19 infection, such as fever, flu, and/or cough, was assessed via the question, “Did you develop fear and worry when you presented with such symptoms as fever, flu, and/or cough during the COVID-19 pandemic?” Those who responded “yes” were asked further questions on what caused them to develop fear when they presented with physical symptoms resembling symptoms of COVID-19 infection. The perception that the area of residence was highly prevalent for COVID-19-positive cases was assessed with the question, “Did you feel that your area of residence was highly prevalent for COVID-19-positive cases?” A history of quarantine because of being a close contact of COVID-19-positive cases was evaluated via the question, “Have you been quarantined for 14 days due to being a close contact of a COVID-19-positive case?” All the responses were grouped as “no” or “yes.” Finally, the average number of working hours in a week during the COVID-19 pandemic was assessed with the question, “How many average working hours did you engage in per week during the COVID-19 pandemic?” The responses were presented as a continuous variable.

#### Depression, Anxiety, and Stress

The severity of depressive, anxiety, and stress symptoms was evaluated by administering the Malay version of the 21-item Depression, Anxiety, and Stress Scale (DASS-21). These symptoms were assessed using three subscales, each consisting of seven items rated using a 4-point Likert scale (0 = Did not apply to me at all to 3 = Applied to me very much or most of the time). The primary outcome is the sum score of each subscale multiplied by 2, in which a higher score represents worse depression, anxiety, and stress symptoms. The total score of each subscale ranged from 0 to 42 (Lovibond and Lovibond, 1995). This tool has been adapted to the Malay language and validated in various clinical and nonclinical populations, demonstrating sound psychometric properties (Cronbach's α ≥ 0.70; Musa et al., 2007; Nur Azma et al., 2014).

#### Perceived Social Support

The respondents' degree of perceived social support was assessed with the Malay version of the Multidimensional Scale of Perceived Social Support (MSPSS). This self-administered questionnaire addresses the perceived adequacy of social support using 12 items designated to three domains (family, friends, and significant others social support); each item is rated on a 7-point Likert scale (1 = Very strongly disagree to 7 = Very strongly agree). The primary outcome of the measure is reported as the sum of total perceived social support (total score ranging from 12 to 84), as well as individual domain scores (domain scores ranging from 4 to 28). The sum of the total scores denotes the degree of perceived social support as low (12–48), moderate (49–68), or high (69–84; Grey et al., 2020). The MSPSS has been translated and validated in Malay-speaking populations, demonstrating evidence of good reliability (Cronbach's α > 0.80, Spearman's rho > 0.75) and validity (Ng et al., 2010).

#### QoL

The degree of QoL of the respondents was measured with the Malay version of the World Health Organization Quality of Life-BREF (WHOQOL-BREF). The WHOQOL-BREF, an abbreviated version of the WHOQOL-100, was conceptualized to cater to the subjective evaluation of individuals' QoL on four domains, namely, physical health, psychological, social relationship, and environmental QoL. It consists of 26 items, with 24 items assessing the domains of the WHOQOL-BREF and two additional items measuring overall QoL and general health. The items are rated on a 5-point Likert scale that evaluates the perceived QoL of the respondents for the past two weeks. This measure has been used widely across multiple populations (Min et al., 2002; Hwang et al., 2003; Lucas-Carrasco et al., 2011; Suárez et al., 2018) and validated in the Malay-speaking population (Cronbach's α = 0.89; Hasanah et al., 2003). The mean score of items in each domain represents the domain score, with a higher score reflecting a higher QoL. The estimated norms of the quality of life domains in the general population are as follows: physical health QoL = 73.5 (SD = 18.1), psychological QoL = 70.6 (SD = 14.0), social relationship QoL = 71.5 (SD = 18.2), and environmental QoL = 75.1 (SD = 13.0; Hawthorne et al., 2006).

#### Statistical Analysis

Analyses were performed using the Statistical Package for Social Sciences (SPSS) version 26 (SPSS 26: SPSS Inc., Chicago, Illinois, USA). Descriptive statistics were computed to report the demographics, clinical factors, COVID-19 factors, psychological experiences, social support, and QoL of the respondents. The frequency and percentage were reported for categorical variables, whereas the mean and standard deviation were reported for continuous variables. The association between demographic and clinical characteristics, psychological experiences, COVID-19-related factors, and degree of perceived social support of the respondents (independent variables) and the QoL domain scores of the WHOQOL-BREF (dependent variables) were evaluated using univariate linear regression and multiple linear regression analyses via the backward selection method. We checked all the multiple linear regression models to ensure that all the assumptions for multiple linear regression had been met as follows:

1. The normal probability plot of the regression standardized residual denoted that all the points lay in a reasonably straight diagonal line from left to right, indicating that the residuals were normally distributed in all the models;
2. The variance inflation factors of all the independent variables were <5, and the tolerance scores of all the independent variables in all the models were above 0.2, indicating no multicollinearity between the independent variables;
3. The Durbin–Watson statistic indicated scores of near 2 in all the models, indicating that the residuals were independent;
4. The scatterplot of the predicted regression standardized value against the regression standardized value obtained in all the models revealed a random array of dots, indicating homoscedasticity of the residuals;
5. The Cook's distance statistics indicated that none of the values of the respondents were more than 1 in all the models, denoting that there was an absence of significant outliers in all the models; and
6. Because all the residuals in the models were normally distributed and homoscedastic, the linearity between all the independent variables and dependent variable was confirmed in all the models.

Statistical significance was set at *p* < 0.05 for all the data analyses.

## Results

### Respondents' Characteristics

Table 1 summarizes the demographic and clinical characteristics, COVID-19-related factors, psychological characteristics, perceived social support, and QoL of the respondents. The mean age of the respondents was 38.55 years (SD = 8.40), and more than three-fourths of the respondents were married (*n* = 301, 77.4%). More than one-fourth had a history of pre-existing medical illnesses, especially hypertension, diabetes mellitus, and bronchial asthma (*n* = 104, 26.7%). The common subjective COVID-19-related stressors experienced by the respondents included fear and worry when developing physical symptoms that resembled COVID-19 infection (*n* = 231, 59.4%), stress resulting from frequent exposure to COVID-19 patients (*n* = 179, 46.0%), loss of daily routine because of COVID-19 (*n* = 115, 29.6%), and stress because of annual leave being frozen (*n* = 68, 17.5%). In addition, most respondents perceived that their area of residence was highly prevalent for COVID-19-positive cases (*n* = 379, 89.7%), while the mean working hours per week among the respondents was 24.60 h (SD = 17.87). The mean depression, anxiety, and stress subscale scores of the respondents were 5.35 (SD = 3.65), 5.37 (SD = 3.34), and 7.68 (SD = 4.63), respectively. The mean perceived family, friends, and significant others social support domain scores were 21.86 (SD = 4.66), 20.97 (SD = 4.50), and 22.01 (SD = 5.33), while the mean total perceived social support score was 65.42 (SD = 12.94), indicating a moderate degree of perceived social support (Grey et al., 2020). The mean physical health, psychological, social relationship, and environmental QoL scores were 74.06 (SD = 15.32), 72.31 (SD = 15.66), 70.87 (SD = 19.67), and 75.48 (SD = 14.65).

**Table 1:** Demographic, personal, clinical characteristics, COVID-19 related factors, psychological characteristics, perceived social support, and quality of life of the respondents.

**Variables**	***N***	**%**
Age (years)	38.55^#^	8.40^$^
Gender:		
Male	107	27.5
Female	282	72.5
Marital status:		
Married	301	77.4
Single/divorced/widowed	88	22.6
Education status:		
Primary or secondary education	41	10.5
Up to tertiary education	348	89.5
Religion:		
Non-Muslim	33	8.5
Muslim	356	91.5
Living condition:		
Live alone	23	5.9
Live with colleagues or friends	25	6.4
Live with family	341	87.7
History of medical illness:		
No	285	73.3
Yes	104	26.7
History of psychiatric illness:		
No	379	97.4
Yes	10	2.6
Worry about family member's health:		
No	345	88.7
Yes	44	11.3
Loss of daily routine during COVID-19:		
No	274	70.4
Yes	115	29.6
Average working hours in a week	24.60^#^	17.87^$^
Stress due to annual leave being frozen during COVID-19:		
No	321	82.5
Yes	68	17.5
Stress due to frequent exposure to COVID-19 patients:		
No	210	54.0
Yes	179	46.0
Fear and worry when developed symptoms which resembles COVID-19 (fever, flu, cough):		
No	158	40.6
Yes	231	59.4
Perception that area of residence was highly prevalent for COVID-19 positive cases:		
No	40	10.3
Yes	379	89.7
History of quarantine for 14 days due to close contact with COVID-19 positive cases:		
No	359	92.3
Yes	30	7.7
DASS-21 depression subscale score	5.35^#^	3.65^$^
DASS-21 anxiety subscale score	5.37^#^	3.34^$^
DASS-21 stress subscale score	7.68^#^	4.63^$^
Family support domain score	21.86^#^	4.66^$^
Friends support domain score	20.97^#^	4.50^$^
Significant others domain score	22.01^#^	5.33^$^
Total social support score	65.42^#^	12.94^$^
Physical health QoL score	74.06^#^	15.32^$^
Psychological QoL score	72.31^#^	15.66^$^
Social relationship QoL score	70.87^#^	19.67^$^
Environmental QoL score	75.48^#^	14.65^$^

# *Mean*,

$ *standard deviation*.

### Predictors of Physical Health QoL Among the Respondents

Table 2 illustrates the association among individual demographic and clinical characteristics, COVID-19-related factors, psychological characteristics, perceived social support, and the WHOQOL-BREF domains score among the respondents. Univariate linear regression analysis indicated that several individual clinical characteristics, COVID-19-related factors, psychological characteristics, and social support variables were significantly associated with physical health QoL among the respondents, as shown in Table 2. However, the multiple linear regression model showed that those with a history of pre-existing medical illness, compared with those without a history of pre-existing medical illness (*B* = −4.718, 95% CI = −7.317 to −2.120, standardized β = −0.136, *p* < 0.001), those who were stressed because annual leave was frozen compared with those who were not stressed because of frozen leave (*B* = −3.556, 95% CI = −6.622 to −0.490, standardized β = −0.088, *p* = 0.023), and greater severity of anxiety (*B* = −0.579, 95% CI = −0.904 to −0.254, standardized β = −0.240, *p* = 0.001) were significantly associated with a lower physical health QoL. In contrast, a greater degree of perceived friends social support (*B* = 0.617, 95% CI = 0.292 to 0.943, standardized β = 0.181, *p* < 0.001) and greater degree of perceived significant others social support (*B* = 0.340, 95% CI = 0.045 to 0.635, standardized β = 0.118, *p* = 0.045) significantly predicted higher physical health QoL. The multiple linear regression model, which presented the association between various independent variables and physical health QoL, is given in Table 3.

**Table 2:** The association between individual demographic, personal, clinical characteristics, COVID-19 related factors, psychological characteristics, perceived social support, and the WHOQoL-BREF domains score among the respondents.

**Variables**	**Physical health QoL**	**Psychological QoL**	**Social relationship QoL**	**Environmental QoL**
	**B (95% CI)**	**B (95% CI)**	**B (95% CI)**	**B (95% CI)**
Age	0.175 (-0.007 to 0.356)	0.363 (0.180 to 0.546)	0.337^*^ (0.105 to 0.569)	0.399^*^ (0.230 to 0.569)
Gender:				
Male	Reference	Reference	Reference	Reference
Female	−1.707 (-5.127 to 1.713)	−2.081 (-5.575 to 1.413)	−2.833 (-7.221 to 1.555)	−1.210 (-4.482 to 2.063)
Marital status:				
Married	Reference	Reference	Reference	Reference
Single/divorced/widowed	−1.416 (-5.068 to 2.236)	−4.620^*^ (-8.327 to−0.913)	−9.703^*^ (-14.294 to−5.111)	−1.749 (-5.240 to 1.742)
Education status:				
Up to secondary education	Reference	Reference	Reference	Reference
Up to tertiary education	−0.667 (-5.647 to 4.313)	−3.412 (-8.490 to 1.667)	−2.407 (-8.796 to 3.983)	1.080 (-3.682 to 5.841)
Religion:				
Non-Muslim	Reference	Reference	Reference	Reference
Muslim	−2.615 (-8.097 to 2.867)	3.389 (-2.210 to 8.988)	2.277 (-4.766 to 9.320)	2.542 (-2.700 to 7.785)
Living condition:				
Live alone/with colleagues/with friends	Reference	Reference	Reference	Reference
Live with family	0.250 (-6.233 6.734	6.156 (-0.442 to 12.754)	9.152^*^ (0.878 to 17.426)	3.558 (-2.632 to 9.748)
History of medical illness:				
No	Reference	Reference	Reference	Reference
Yes	−6.305^*^ (-9.305 to−2.908)	−1.478 (-5.006 to 2.050)	−5.271^*^ (-9.676 to−0.866)	−0.403 (-3.707 to 2.901)
History of psychiatric illness:				
No	Reference	Reference	Reference	Reference
Yes	−23.260^*^ (-32.639 to−13.881)	−28.547^*^ (-38.002 to−19.093)	−29.120^*^ (-41.180 to−17.060)	−14.552^*^ (-23.678 to−5.427)
Worry about family member's health:				
No	Reference	Reference	Reference	Reference
Yes	−1.248 (-6.075 to 3.578)	−4.787 (-9.698 to 0.124)	−10.054^*^ (-16.171 to−3.937)	−0.795 (-5.412 to 3.821)
Loss of daily routine during COVID-19:				
No	Reference	Reference	Reference	Reference
Yes	−6.754 (-10.037 to−3.472)	−9.099^*^ (-12.401 to−5.797)	−7.027^*^ (-11.272 to−2.782)	−6.197^*^ (-9.342 to−3.053)
Average working hours in a week	−0.053 (-0.138 to 0.033)	−0.094^*^ (-0.181 to−0.007)	−0.093 (-0.202 to 0.017)	−0.080 (-0.161 to 0.002)
Stress due to annual leave being frozen during COVID-19:				
No	Reference	Reference	Reference	Reference
Yes	−8.272^*^ (-12.213 to−4.332)	−9.219^*^ (-13.230 to−5.209)	−10.911^*^ (-15.964 to−5.858)	−7.441^*^ (-11.219 to−3.663)
Stress due to frequent exposure to COVID-19 patients:				
No	Reference	Reference	Reference	Reference
Yes	−4.482^*^ (-6.504 to−2.460)	−4.362^*^ (-6.433 to−2.291)	−4.362^*^ (-6.984 to−1.739)	−4.520^*^ (-6.448 to−2.592)
Fear and worry when developed symptoms which resembles COVID-19 (fever, flu, cough):				
No	Reference	Reference	Reference	Reference
Yes	−2.119 (-4.248 to 0.010)	−1.175 (-3.358 to 1.009)	−0.870 (-3.616 to 1.876)	−1.258 (-3.301 to 0.784)
Perception that area of residence was highly prevalent for COVID-19 positive cases:				
No	Reference	Reference	Reference	Reference
Yes	−2.816^*^ (-4.706 to−0.926)	−7.567^*^ (-12.657 to−2.477)	−12.842^*^ (-19.178 to−6.506)	−5.939^*^ (-10.717 to−1.161)
History of quarantine for 14 days due to close contact with COVID-19 positive cases:				
No	Reference	Reference	Reference	Reference
Yes	−3.318 (-9.040 to 2.405)	−3.302 (-9.151 to 2.548)	−3.870 (-11.219 to 3.479)	−1.457 (-6.937 to 4.023)
DASS-21 depression subscale score	−1.303^*^ (-1.493 to−1.114)	−1.448^*^ (-1.634 to−1.263)	−1.584^*^ (-1.834 to−1.335)	−1.080^*^ (-1.272 to−0.889)
DASS-21 anxiety subscale score	−1.351^*^ (-1.551 to−1.150)	−1.282^*^ (-1.493 to−1.071)	−1.452^*^ (-1.726 to−1.178)	−0.967^*^ (-1.176 to−0.757)
DASS-21 stress subscale score	−1.134^*^ (-1.300 to−0.969)	−1.177^*^ (-1.345 to−1.009)	−1.318^*^ (-1.539 to−1.097)	−0.829^*^ (-1.002 to−0.656)
Family support domain score	1.350^*^ (1.050 to 1.649)	1.733^*^ (1.445 to 2.021)	1.949^*^ (1.575 to 2.323)	1.459^*^ (1.181 to 1.738)
Friends support domain score	1.426^*^ (1.116 to 1.735)	1.776^*^ (1.476 to 2.075)	2.079^*^ (1.694 to 2.464)	1.574^*^ (1.288 to 1.859)
Significant others domain score	1.035^*^ (0.767 to 1.304)	1.463^*^ (1.208 to 1.718)	2.040^*^ (1.732 to 2.347)	1.183^*^ (0.935 to 1.431)

* *p < 0.05*.

**Table 3:** Multiple linear regression analysis with backward selection method between demographic and personal characteristics, COVID-19 related factors, psychological characteristics, perceived social support, and physical health QoL among the respondents.

**Variables**	**β (95% CI)**	**Standardized β**	***p*-value**
Marital status:			
Married	Reference		
Single/divorced/widowed	2.837 (-0.157 to 5.831)	0.078	0.063
History of medical illness:			
No	Reference		
Yes	−4.718 (-7.317 to−2.120)	−0.136	<0.001^*^
Stress due to annual leave being frozen during COVID-19:			
No	Reference		
Yes	−3.556 (-6.622 to−0.490)	−0.088	0.023^*^
DASS-21 depression subscale score	−0.306 (-0.642 to 0.030)	−0.133	0.074
DASS-21 anxiety subscale score	−0.579 (-0.904 to−0.254)	−0.240	0.001^*^
DASS-21 stress subscale score	−0.307 (-0.622 to 0.008)	−0.153	0.056
Friends support domain score	0.617 (0.292 to 0.943)	0.181	<0.001^*^
Significant others domain score	0.340 (0.045 to 0.635)	0.118	0.045^*^

* *Statistical significance at p < 0.05; multiple regression analysis included all demographic, personal characteristics, COVID-19 related factors, psychological characteristics, and perceived social support as independent variables, and then backward selection method was applied to remove non-significant variables*.

### Predictors of Psychological QoL Among the Respondents

Univariate linear regression analysis revealed that several demographic and COVID-19-related factors, psychological characteristics, and social support variables were significantly associated with psychological QoL (Table 2). The multiple linear regression model indicated that only a few COVID-19-related, psychological, and social support variables were significantly associated with psychological QoL. Those who were stressed because of loss of daily routine compared with those who were not stressed from a loss of daily routine (*B* = −3.433, 95% CI = −5.922 to −0.944, standardized β = −0.100, *p* = 0.007), those who were stressed because of annual leave being frozen compared with those who were not stressed because of frozen leave (*B* = −3.043, 95% CI = −5.999 to −0.086, standardized β = −0.074, *p* = 0.044), greater severity of depression (*B* = −0.612, 95% CI = −0.918 to −0.305, standardized β = −0.260, *p* < 0.001), and greater severity of stress (*B* = −0.409, 95% CI = −0.668 to −0.151, standardized β = −0.200, *p* = 0.002) were significantly associated with lower psychological QoL. In contrast, a greater degree of perceived friends social support (*B* = 0.757, 95% CI = 0.457 to 1.058, standardized β = 0.217, *p* < 0.001) and a greater degree of perceived significant others social support (*B* = 0.603, 95% CI = 0.348 to 0.858, standardized β = 0.205, *p* < 0.001) significantly predicted higher psychological QoL. The multiple linear regression model, which presented the association between various independent variables and psychological QoL, is summarized in Table 4.

**Table 4:** Multiple linear regression analysis with backward selection method between demographic and personal characteristics, COVID-19 related factors, psychological characteristics, perceived social support, and psychological QoL among the respondents.

**Variables**	**β (95% CI)**	**Standardized β**	***p*-value**
Loss of daily routine during COVID-19:			
No	Reference		
Yes	−3.433 (-5.922 to−0.944)	−0.100	0.007^*^
Stress due to annual leave being frozen during COVID-19:			
No	Reference		
Yes	−3.043 (-5.999 to−0.086)	−0.074	0.044^*^
DASS-21 depression subscale score	−0.612 (-0.918 to−0.305)	−0.260	<0.001^*^
DASS-21 stress subscale score	−0.409 (-0.668 to−0.151)	−0.200	0.002^*^
Friends support domain score	0.757 (0.457 to 1.058)	0.217	<0.001^*^
Significant others domain score	0.603 (0.348 to 0.858)	0.205	<0.001^*^

* *statistical significance at p < 0.05; multiple regression analysis with backward included all demographic, personal characteristics, COVID-19 related factors, psychological characteristics, and perceived social support as independent variables, and then backward selection method was applied to remove non-significant variables*.

### Predictors of Social Relationship QoL Among the Respondents

Univariate linear regression analysis showed that several demographic, clinical, COVID-19 related, psychological, and social support variables were significantly associated with social relationship QoL (Table 2). However, the multiple linear regression model pinpointed that only a few COVID-19-related, psychological, and social support variables were significantly associated with social relationship QoL. Those who were stressed because of annual leave being frozen compared with those who were not stressed because of frozen leave (*B* = −3.640, 95% CI = −6.895 to −0.385, standardized β = −0.082, *p* = 0.029), greater severity of depression (*B* = −0.429, 95% CI = −0.832 to −0.027, standardized β = −0.145, *p* = 0.037), and greater severity of stress (*B* = −0.595, 95% CI = −0.936 to −0.254, standardized β = −0.231, *p* = 0.001) were significantly likely to have lower social relationship QoL. In contrast, a greater degree of perceived friends social support (*B* = 0.717, 95% CI = 0.310 to 1.125, standardized β = 0.164, *p* = 0.001) and a greater degree of perceived significant others social support (*B* = 1.169, 95% CI = 0.820 to 1.519, standardized β = 0.317, *p* < 0.001) significantly predicted higher social relationship QoL. The multiple linear regression model, which presented the association between various independent variables and social relationship QoL, is presented in Table 5.

**Table 5:** Multiple linear regression analysis with backward selection method between demographic and personal characteristics, COVID-19 related factors, psychological characteristics, perceived social support, and social relationship QoL among the respondents.

**Variables**	**β (95% CI)**	**Standardized β**	***p*-value**
Worry about family member's health:			
No	Reference		
Yes	−4.487 (-9.233 to 0.259)	−0.072	0.064
History of medical illness:			
No	Reference		
Yes	−3.813 (-7.642 to 0.017)	−0.074	0.051
Stress due to annual leave being frozen during COVID-19:			
No	Reference		
Yes	−3.640 (-6.895 to−0.385)	−0.082	0.029^*^
DASS-21 depression subscale score	−0.429 (-0.832 to−0.027)	−0.145	0.037^*^
DASS-21 stress subscale score	−0.595 (-0.936 to−0.254)	−0.231	0.001^*^
Friends support domain score	0.717 (0.310 to 1.125)	0.164	0.001^*^
Significant others domain score	1.169 (0.820 to 1.519)	0.317	< 0.001^*^

* *statistical significance at p < 0.05; multiple regression analysis with backward included all demographic, personal characteristics, COVID-19 related factors, psychological characteristics, and perceived social support as independent variables, and then backward selection method was applied to remove non-significant variables*.

### Predictors of Environmental QoL Among the Respondents

Univariate linear regression analysis revealed that several demographic, clinical, COVID-19-related, psychological, and social support variables were significantly associated with environmental QoL (Table 2). The multiple linear regression model indicated that only a few demographic characteristics, COVID-19-related factors, and psychological and social support variables were significantly associated with environmental QoL. Those who were stressed because of annual leave being frozen compared with those who were not stressed because of frozen leave (*B* = −2.018, 95% CI = −3.665 to −0.371, standardized β = −0.102, *p* = 0.016) and those with a greater severity of depression (*B* = −0.449, 95% CI = −0.731 to −0.167, standardized β = −0.204, *p* = 0.002) were significantly likely to have lower environmental QoL. Conversely, increasing age (*B* = 0.268, 95% CI = 0.126 to 0.410, standardized β = 0.154, *p* < 0.001); being single, divorced, or widowed compared with being married (*B* = 4.679, 95% CI = 1.594 to 7.764, standardized β = 0.134, *p* = 0.003); greater degree of perceived friends social support (*B* = 0.755, 95% CI = 0.432 to 1.079, standardized β = 0.232, *p* < 0.001); and greater degree of perceived significant others social support (*B* = 0.601, 95% CI = 0.310 to 0.893, standardized β = 0.219, *p* < 0.001) significantly predicted higher environmental QoL. The multiple linear regression model, which presented the association between various independent variables and environmental QoL, is presented in Table 6.

**Table 6:** Multiple linear regression analysis with backward selection method between demographic and personal characteristics, COVID-19 related factors, psychological characteristics, perceived social support, and environmental QoL among the respondents.

**Variables**	**β (95% CI)**	**Standardized β**	***p*-value**
Age	0.268 (0.126 to 0.410)	0.154	<0.001^*^
Marital status:			
Married	Reference		
Single/divorced/widowed	4.679 (1.594 to 7.764)	0.134	0.003^*^
Religion:			
Non-Muslim	Reference		
Muslim	3.727 (-0.389 to 7.842)	0.071	0.076
Stress due to frequent exposure to COVID-19 patients:			
No	Reference		
Yes	−2.018 (-3.665 to−0.371)	−0.102	0.016^*^
DASS-21 depression subscale score	−0.449 (-0.731 to−0.167)	−0.204	0.002^*^
DASS-21 anxiety subscale score	−0.273 (-0.556 to 0.010)	−0.118	0.059
Friends support domain score	0.755 (0.432 to 1.079)	0.232	<0.001^*^
Significant others domain score	0.601 (0.310 to 0.893)	0.219	<0.001^*^

* *Statistical significance at p < 0.05; multiple regression analysis included all demographic, personal characteristics, COVID-19 related factors, psychological characteristics, and perceived social support as independent variables, and then backward selection method was applied to remove non-significant variables*.

## Discussion

The present study assessed QoL and its predictive factors among healthcare workers in university hospitals and healthcare facilities that accommodated COVID-19 cases during the post-movement lockdown phase. As a comparison with the norm of the physical health QoL in the general population in non-pandemic settings (73.5, SD = 18.1; Hawthorne et al., 2006), the respondents in our study exhibited no decrease in physical health QoL (74.06, SD = 15.3) during the COVID-19 pandemic. Despite the absence of deterioration of physical health QoL among the healthcare workers in this study, it is worth noting that clinical factors (history of pre-existing medical illness), COVID-19-related stressors (annual leave being frozen), and psychological sequelae of COVID-19 (greater severity of anxiety symptoms) could impair the physical health QoL of healthcare workers during the COVID-19 pandemic. A large proportion of respondents in this study presented with a history of pre-existing medical illness, such as diabetes mellitus, hypertension, chronic pain, and bronchial asthma. A study on primary healthcare workers in Brazil also reported that respondents presenting with negative self-perceived health status or being diagnosed with systemic illnesses by physicians contributed to poor physical health QoL (Teles et al., 2014). Moreover, a study on how chronic back pain affects the QoL of a cohort of medical workers also indicated that a greater degree of disability due to the medical condition was associated with poor physical health QoL (Mroczek et al., 2020). Hence, our findings further strengthened the reciprocal relationship between pre-existing medical conditions and poor physical health QoL.

Our findings highlighted the importance of having ample time off from clinical duties among healthcare workers during the COVID-19 pandemic because not being permitted to take leave diminished physical health QoL, and this effect on QoL could be related to burnout. Burnout among healthcare personnel during the COVID-19 pandemic has been well documented; moreover, burnout has been reported to impair the QoL of healthcare workers (Amanullah and Shankar, 2020; Çelmeçe and Menekay, 2020). Consistent with the results from previous studies of the QoL of healthcare workers, our findings also indicated that increased severity of anxiety and a higher degree of work environment stressors disrupted the QoL of healthcare workers during the COVID-19 pandemic (Çelmeçe and Menekay, 2020; Suryavanshi et al., 2020). Reciprocal relationships between a higher severity of anxiety, poor sleep quality, and lower health-related QoL have been documented in healthcare professionals who are directly involved in treating COVID-19 patients (Stojanov et al., 2020). In fact, patients with anxiety disorders, such as panic disorder, generalized anxiety disorder, panic disorder with agoraphobia, and social phobia—which present with a number of physical symptoms during an anxiety attack—have lower physical health-related QoL compared with the norm in the general population (Beard et al., 2010).

The psychological QoL of the healthcare workers in this study (72.3, SD = 15.7) was shown to be comparable to that of the general population in a non-pandemic setting (70.6, SD = 14.0). Notwithstanding, COVID-19-related factors (e.g., stress from loss of daily routine and stress due to annual leave being frozen) and psychological complications (greater severity of depression and stress symptoms) contributed to lowering of psychological QoL. In our study, further questioning of respondents revealed that they felt frustrated with the loss of daily routine (e.g., engaging in leisure and sporting activities, vacation with family, visiting their elderly parents in their hometown, praying in places of worship, and spending time with friends in the cafeteria, which helped them neutralize their work stress). Moreover, the need to work extra hours with little break because of annual leave being frozen aggravated their stress and hampered their chance to distress from their highly demanding working schedules. Working long hours without ample leave or sufficient breaks has been reported to induce burnout among healthcare workers and may contribute to diminishing psychological QoL (Asante et al., 2019; Roslan et al., 2021). Healthcare workers who work in COVID-19-designated units or hospitals encounter persistent increases in patient load while facing other issues, such as insufficient numbers of working medical professionals, shortages of personal protective equipment and resources for critical care, as well as an increasing number of clinicians being infected and resulting in mortality in some instances, greatly increasing their risk of unmanageable stress and depression (Cai et al., 2020a; Lu et al., 2020; Manh Than et al., 2020). Depression contributes to a lower psychological QoL (Shumye et al., 2019), and it has been reported to lower the QoL of healthcare workers during the COVID-19 pandemic, particularly in terms of the mental health component (An et al., 2020; Stojanov et al., 2020). These factors explained the link between stress from loss of daily routine, stress because of annual leave being frozen, greater severity of depression and stress symptoms, and lower psychological QoL in our study.

The social relationship QoL of the respondents in this study (70.9, SD = 19.7) was slightly lower than the norm in the general population in a non-pandemic setting (71.5, SD = 18.2). Movement restrictions imposed during the outbreak as an effort to break the chain of infection may have limited the social interaction between the healthcare workers and their social circle. Changes in the pattern of social interactions and concerns among the general population have been documented after the emergence of the COVID-19 pandemic. Concerns regarding leisure activities and friends decreased, whereas concerns regarding health, family, and religion increased after the emergence of the pandemic (Li et al., 2020b). This may explain the slightly lower social relationship QoL among healthcare workers in this study compared with the general population in a non-pandemic setting. In addition, we found that COVID-19-related factors (e.g., stress due to annual leave being frozen) and psychological complications (greater severity of depression and stress symptoms) contributed to lowering of social relationship QoL. The reciprocal relationship between annual leave being frozen and lowered social relationship QoL could be deduced from the decreased ability to engage in social relationships resulting from a lack of time for interaction with family and social circle of healthcare workers because of longer working hours during the pandemic. In addition, a reduction in social interaction with classmates and family members has been reported in response to the COVID-19 pandemic beyond the social distancing implemented to curb the spread of the infection (Chou et al., 2020). The impact of depression and stress on social functioning is well documented, and increased severity of stress and depression may have further aggravated social functional impairment during the COVID-19 pandemic (Kupferberg et al., 2016; Gallagher et al., 2020). In fact, COVID-19-induced social functional impairment is strongly associated with depression and poor psychological wellbeing (Dawel et al., 2020). Hence, the finding that greater severity of depression and stress in our study predicted lower social relationship QoL was consistent with the findings of previous studies (Dawel et al., 2020; Vafaei et al., 2020).

There was no reduction of environmental QoL among the respondents in our study (75.5, SD = 14.7) compared with the norm of the general population in the non-pandemic state (75.1, SD = 13.0; Hawthorne et al., 2006). The two following factors were associated with lower environmental QoL among the respondents: stress because of frequent exposure to COVID-19 patients and greater severity of depressive symptoms. The environmental QoL domain of the WHOQOL-BREF assesses respondents' perceived satisfaction regarding the safety and health condition of the surrounding environment, financial status, availability of information, availability of recreational activities, satisfaction with place of living, availability of healthcare, and availability of transportation (Hawthorne et al., 2006). Healthcare workers who felt stressed because of frequent exposure to COVID-19, patients may have felt that their surrounding environment was unsafe and the health condition of their surroundings was compromised; they may also have experienced reduced involvement in recreational activities. This situation may have led to a lowering of environmental QoL. Similar to our finding, it has been reported that higher severity of depression to predict lower environmental QoL in the general population in the non-pandemic context. Nevertheless, depression is associated with a lesser lowering effect on environmental QoL compared with its effect on physical health and psychological QoL (González-Blanch et al., 2018).

Demographic characteristics, such as increasing age; being single, divorced, or widowed; and higher perceived social support from friends and significant others predicted higher environmental QoL among the respondents in this study. Healthcare workers of older age in our study were found to have higher perceived safety and health security of the surrounding environment despite working in a high-risk context. Senior healthcare workers may have more experience working in clinical settings, especially in the management of infectious epidemics and pandemics, and they may have acquired higher skill competency compared with their younger counterparts. This is consistent with recent findings highlighting better adaptation to the COVID-19 pandemic among experienced healthcare workers who were previously exposed to SARS outbreaks (Cai et al., 2020b; Song et al., 2020). Better adaptation among experienced healthcare workers is indicated by better control while working in a high-risk environment, being well equipped with previous experience of preventive measures to protect oneself, less worry in handling patients with COVID-19-related symptoms, and higher resilience during this pandemic (Shanafelt and Noseworthy, 2017; Smith et al., 2020). Stojanov's et al. (2020) study on health-related QoL of healthcare workers during the COVID-19 reported a significant but weak positive correlation between being married and health-related QoL. Surprisingly, those who were single, divorced, or widowed in this study had significantly higher environmental QoL compared with those who were married. One possible explanation for this could be the higher perceived safety of the surrounding environment among those who were single, divorced, or widowed and lived. When asked, most of the respondents indicated that they were worried about spreading the infection to their family members. Hence, for those who are single, divorced, or widowed live alone, this may reduce the risk of spreading the infection to their family members, resulting in higher perceived safety of the surrounding environment.

It is interesting to find that the support received from friends and significant others, rather than family support, predicted higher scores in all the domains of QoL (physical health, psychological, social relationship, and environmental QoL). Greater social support buffers the effect of individual stress perception, facilitates positive coping strategies, and reduces the negative impact of stress on physical and mental health, contributing to higher QoL among healthcare workers. In fact, better social relations and social support have been documented to predict higher psychological and social relationship QoL among healthcare workers (Sun et al., 2017; Asante et al., 2019).

Evidence has established a link between social support adequacy (e.g., friends, spousal, or partner social support) and a lower risk of morbidity (e.g., cardiovascular disease and hypertension; Coffey and Coleman, 2001; Umberson and Montez, 2010; Steptoe and Kivimäki, 2013). Despite the negative impact of COVID-19-related stress on healthcare workers, having supportive co-workers and superiors facilitates better health adaptation in response to the changes experienced during the uncertain time of the COVID-19 pandemic (Zhang et al., 2020b). Hence, a greater degree of perceived support from friends and significant others will enhance the physical health QoL of healthcare workers. As healthcare workers were required to work extra hours, taking care of an increasing number of COVID-19 patients, and as their time spent with friends and colleagues increased while time with family reduced, their immediate source of social support would derive from friends and colleagues working in the same facility. Qualitative studies on the mental health status of healthcare workers during the pandemic have identified the important role of social support from friends, colleagues, immediate supervisors, patients, and significant others to overcome emotional barriers to quality care of patients, with such barriers including depression, anxiety, and stress resulting from the uncertainty of the risk of being infected, as well as the need to abide by new norms and protective measures (Liu et al., 2020; Zhang et al., 2020c). Moreover, the respondents answers revealed that the sources of social interaction, social networking, and social support of the healthcare workers in our study were friends and colleagues instead of family during the COVID-19 pandemic. It is no surprise that increased social support from friends and significant others among the respondents in our study was essential to enhancing their psychological and social relationship QoL. Similarly, increasing social interaction and social networking with friends and colleagues related to more time spent in the healthcare facilities they worked in may have increased perceived safety in the surrounding environment and the sharing of information. This potentially resulted in the increased environmental QoL found among the healthcare workers in our study.

Based on our findings, we highlighted the salient roles of COVID-19-related stressors, psychological sequelae, and social support in predicting the QoL of healthcare workers in our study. COVID-19-related stressors (e.g., stress due to annual leave being frozen, loss of daily routine, and frequent exposure to COVID-19 patients) and psychological sequelae (e.g., greater severity of depression, anxiety, and stress) contributed to lower QoL. Conversely, greater perceived social support received from friends and significant others predicted higher QoL among healthcare workers. In addition, clinical and demographic characteristics predicted QoL to a lesser extent; a history of pre-existing medical illness was only associated with lower physical health QoL, whereas older age and being single, divorced, or widowed were only predictive of higher environmental QoL.

In essence, there were a few implications that could be derived from our findings to safeguard QoL among healthcare workers. Considering the COVID-19-related stressors experienced by healthcare workers, authorities should ensure that sufficient personal protective equipment and efficient standard operating procedures and steps to prevent the spread of infection while working in healthcare facilities are carried out. Knowing that psychological sequelae are rising among healthcare workers during the uncertain time of the COVID-19 pandemic, sufficient psychological services for healthcare workers should be provided to safeguard their mental wellbeing. A self-help stress management manual, online psychotherapy, or group psychosocial intervention (e.g., mindfulness-based stress reduction) should be made easily accessible for healthcare workers. Given that our study highlighted the pivotal role of social support from friends and colleagues in enhancing the QoL of healthcare workers, psychosocial interventions that focus on the themes of communication and team building, workload and time management, and leadership development may facilitate social support and improved mental wellbeing among healthcare workers in the workplace (Gray et al., 2019).

Several study limitations are worth mentioning. First, this study offers a snapshot of healthcare workers' adaptation to the pandemic during the post-MCO phase, as reflected in their QoL. However, the findings could not determine whether this adaptation can change over time because this study utilized a cross-sectional design. Although we identified the salient roles of COVID-19-related stressors, psychological sequelae, and social support in determining the QoL of healthcare workers, we did not collect prospective data on QoL during and after movement lockdown to allow comparison of QoL to be assessed between the two different moments. Second, the generalizability of the findings was limited by the adoption of snowball sampling for subject recruitment in the interest of maximizing the number of respondents because the study was conducted online. Moreover, the mean age of the respondents in our study was 38.55 years, which was not representative of the entire healthcare worker population in Malaysia, affecting the generalizability of the findings. Third, the study was conducted using self-reported questionnaires, which may have led to under- or over-estimation of the psychological experience of the respondents. Fourth, we did not include data on the proportion of healthcare workers directly involved in the care of COVID-19 patients or data on the departments/units in which they worked. These may be confounding factors that affect the QoL of healthcare workers. Finally, the responses to COVID-19-related factors were measured only as binary coding of the “yes/no” response, which may have limited the richness or amount of information obtained.

To conclude, this study outlined how the QoL of university-based healthcare workers was affected by an infection pandemic, and particularly, it showed that the only domain of QoL that raised concern was social relationship QoL. Interestingly, COVID-19-related stressors, psychological sequelae in response to the COVID-19 pandemic, and social support largely predicted all QoL domains for those who worked in clinical settings. This highlights the salient role of these three categories of psychosocial factors for a successful adaptation to the new norms of the pandemic among healthcare workers. Clinically, to improve the QoL of healthcare workers who presented with psychological complications related to COVID-19, it is vital that treating mental health providers screen for predisposing COVID-19-related stressors and a history of pre-existing medical illness, as well as to provide psychosocial intervention in the form of group therapy to enhance social support among healthcare workers. To confirm our findings, we recommend that future studies employ a longitudinal study design with a mixed method of assessment to further evaluate the effects of COVID-19-related stressors on QoL and also to investigate whether social support, particularly perceived social support from friends and significant others, would moderate the effects of COVID-19-related stressors and psychological sequelae on QoL of healthcare workers.

## Data Availability Statement

The original contributions presented in the study are included in the article, further inquiries can be directed to the corresponding author.

## Ethics Statement

The studies involving human participants were reviewed and approved by Human Research Ethics Committee USM, Division of Research & Innovation (R&I), USM Health Campus, 16150 Kubang Kerian, Kelantan, Malaysia; and the Medical Research Committee of the Faculty of Medicine, Universiti Kabangsaan Malaysia, 56000 Cheras, Kuala Lumpur, Malaysia. The patients/participants provided their written informed consent to participate in this study.

## Author Contributions

ML and LW conceptualized and design the study. ML, LW, NM, ST, and MM involved in data collection. ML and NM involved in data and statistical analysis. ML wrote the first draft of the manuscript. All authors involved in the revision of the manuscript and approved the submitted version.

## Conflict of Interest

The authors declare that the research was conducted in the absence of any commercial or financial relationships that could be construed as a potential conflict of interest.
